# ELISA based assays to measure adenosine deaminases concentration in serum and saliva for the diagnosis of ADA2 deficiency and cancer

**DOI:** 10.3389/fimmu.2022.928438

**Published:** 2022-07-28

**Authors:** Wenwen Luo, Liang Dong, Fenghong Chen, Wenbin Lei, Liya He, Qing Zhou, Thierry Lamy, Andrey V. Zavialov

**Affiliations:** ^1^ Guangzhou Institute of Pediatrics, Guangzhou Women and Children’s Medical Center, Guangzhou Medical University, Guangzhou, China; ^2^ Otorhinolaryngology Hospital, The First Affiliated Hospital of Sun Yat-sen University, Guangzhou, China; ^3^ Life Sciences Institute, Zhejiang University, Hangzhou, China; ^4^ Liangzhu Laboratory, Zhejiang University Medical Center, Hangzhou, China; ^5^ Department of Hematology, Pontchaillou University Hospital, Rennes, France; ^6^ Turku Centre for Biotechnology, University of Turku, Turku, Finland

**Keywords:** adenosine deaminase 2, ADA2 deficiency, head and neck cancer, large granular leukocyte leukemia, adenosine deaminase

## Abstract

Adenosine deaminases (ADAs) are enzymes of purine metabolism converting adenosine to inosine. There are two types of ADAs in humans ADA1 and ADA2. While both ADA1 and ADA2 share the same substrate, they differ in expression, cellular localization, and catalytic properties. The genetic deficiency of ADA1 results in severe combined immunodeficiency (SCID), while lack in ADA2 (DADA2) results in multiple phenotypes ranging from systemic inflammation to vascular pathology. Clinical studies have shown that the levels of ADAs in biological fluids are altered in pathophysiological conditions, suggesting that ADA activity could be a convenient marker for the diagnosis of immune diseases and cancer. Here, we describe sensitive and straightforward ELISA assays to measure ADA1 and ADA2 concentrations in biological fluids. Analysis of the serum and saliva samples from the healthy controls and DADA2 patients revealed that ADA2 enzyme concentration is significantly lower in patients than in healthy controls. In contrast, the concentration of ADA2 increases in the serum of patients with large granular leukocyte leukemia (LGLL) and patients’ saliva with head and neck cancer. Thus, this simple, non-invasive method allows for distinguishing healthy controls from the affected patient. It can be implemented in screening and diagnosis of DADA2 and follow up the treatment of LGLL and several types of head and neck cancer.

## Introduction

It has been shown that adenosine plays an essential role in regulating immune responses. Adenosine accumulates at inflammation and tumor growth sites and binds to adenosine receptors expressed by immune cells in response to activation signals (1). Adenosine deaminases decrease the local concentration of adenosine. Two enzymes, ADA1 and ADA2, with adenosine deaminase activity were found in serum and other biological fluids (2). ADA1 is primarily an intracellular enzyme that breaks down adenosine and reduces the concentration of the adenosine derivatives that are toxic to immune cells. It has been found that mutations in the ADA1 gene lead to severe combined immunodeficiency characterized by lack of antibody production and lower numbers of T and B cells (3). Although the concentration of ADA1 is elevated in different pathologies, the mechanism of the enzyme secretion from cells is unknown (4). The high level of ADA1 in biological fluids could be explained by the enzyme release from the dying cells in the sites of inflammation of tumor growth. In contrast to ADA1, ADA2 is a secreted protein constantly expressed mainly by cells of the myeloid lineage, such as macrophages and dendritic cells (5). Activated T cells infected with HIV-1 can also be a source of ADA2 in patients with HIV (6, 7). Interestingly, ADA2 has at least 80 times lower Km than ADA1 (**Table 1**), suggesting that ADA2 may have another function apart from its ADA activity (8). Patients with mutations in the gene encoding ADA2 (DADA2 patients) display multiple health problems such as early-onset systemic inflammation, multiple ischemic strokes, panlymphopenia, and hypogammaglobulinemia, suggesting a role of ADA2 in the immune system regulation (9–11).

**Table 1:** Catalytic parameters of recombinant human ADA2 and ADA1.

	K_m_	pH optimum	ADA activity corresponding to 1 ng/ml
ADA1	26 µM	7.3	3.25 (IU/l)
ADA2	2.2 mM	6.8	1.1 (IU/l)

Multiple clinical studies have shown that the activity of ADA2 increases in response to intracellular pathogens and cancers. Therefore, detecting the levels and activity of ADA2 is a sensitive, cheap, and non-invasive way to diagnose often symptomless chronic diseases. For a long time, the enzyme’s activity has been used as a specific marker for tuberculosis in pleural fluids (12). The concentration of ADA2 in serum is increased in several inflammatory disorders and cancers, especially blood cancers. Therefore, ADA2 could be a general marker for immune system activation. Thus, the level of ADA2 in serum could be used as a predictive marker and management of HIV and AIDS (7, 13, 14), active tuberculosis (15), juvenile rheumatoid arthritis and systemic lupus erythematosus (16), macrophage activation syndrome in systemic juvenile idiopathic arthritis (17), liver fibrosis in nonalcoholic fatty liver disease (18), breast cancer (19), adult T-cell leukemia (7), and ovarian tumor (20). The decrease in ADA2 concentration in serum has been reported for patients with chronic heart failure (21)

It was recently found that ADA2 is a prognostic factor associated with prolonged cancer patient survival. It was proposed that PEGylated ADA2 could inhibit tumor growth by decreasing the extracellular concentration of adenosine (22). Another study has shown that the decrease in ADA2 activity in serum of patients with breast, gastric, colon cancer, and lymphoma, in particular, correlates with the effectiveness of the cancer treatment. It was suggested that the measurement of plasma AD2 before and after the first dose of chemotherapy could predict the drug’s efficacy (23).

The use of ADA2 as a marker of immune disorders and cancer in saliva has not been fully explored. The only study on ADA2 in saliva shows that the enzyme activity is increased in saliva in patients with coronavirus infection COVID-19 (24). However, a significant problem in determining the levels and activity of ADA2 is the presence of ADA1 in biological fluids. The absence of a specific ADA2 inhibitor and the difference in the catalytic parameters between ADA2 and ADA1 affect the accuracy and make it hard to compare the results of the studies utilizing different enzymatic assays. In addition, the presence of bacterial ADA should also be considered while analyzing ADA activity in saliva. In this study, we solve these problems by using ADA2-specific polyclonal antibodies to isolate ADA2 from these different proteins and use enzymatic and non-enzymatic methods to detect ADA2 coupled to its specific antibody. We describe and evaluate new strategies for the quantitative detection of ADA2 in biological samples. We analyze serum and saliva samples to demonstrate their utility in diagnosing DADA2, LGLL, and head and neck cancers using these assays.

## Materials and methods

### Study design and subjects

The study was approved by the local Ethical Research Committee. The serum samples with LGLL were kindly provided by Dr.Thierry Lamy from the Department of Hematology, Pontchaillou University Hospital, Rennes, France. Dr. Zhou Qing from Zhejiang University provided saliva samples from patients with a confirmed diagnosis of DADA2. The saliva samples of healthy children and children with respiratory diseases and tonsillitis were obtained from Dr. Liya He, Guangzhou Women and Children’s Medical Center. The group consisted of kids matched for age to the ADA2 patients. The adult control group consisted of healthy people over 18 years old. The sample was collected from the Guangzhou Institute of Pediatrics students and staff. The saliva samples of head and neck cancer patients were obtained in Otorhinolaryngology Hospital, The First Affiliated Hospital of Sun Yat-sen University. This project excludes the participants with fixed orthodontics, oral drug treatments, gingival overgrowth, and diagnosed with psychomotor disorders. All of the HD are tested negative for the nucleic acid of COVD-19. All the participants were informed and consented to participate in the trial. The study was carried out following the guidelines.

### Saliva collection and storage

A standard protocol was used for all the participants. Until analysis, unstimulated basal saliva samples were collected and stored at − 20°C. A detailed protocol for saliva sampling and processing is outlined in **Figure 3E**.

### Recombinant proteins and antibodies

Recombinant unmodified ADA2 and ADA1 were overexpressed in 293T cells and purified as described previously (25, 26). Rabbits were immunized 2 times with 1 mg of recombinant ADA2. The polyclonal antibodies were purified from the rabbit serum using a HiTrap protein G antibody purification column (Cytiva) according to the manufacturer’s protocol. Further, Recombinant ADAs and affinity-purified anti-ADA2 antibodies were chemically biotinylated using EZ-Link™ NHS-PEG4-Biotin (ThermoFicher). The anti-ADA2 antibodies were purified on a HiTrap Streptavidin column loaded with biotinylated ADA2.

### Enzyme-linked immunosorbent assay (ELISA) for measurement of the active ADA2 concentration

ELISA plates (Greiner Bio-One) were coated overnight at 4°C with 100 µl of 5 µg/ml rabbit anti-ADA2 polyclonal antibodies in PBS with 0.02% NaN_3_. After washing the plates 3 times with 200 µl PBS-Tween 20 buffer and blocking with 2% BSA in PBS with 0.02% NaN_3_ for 1 hour, 100 µl of recombinant ADA2 standards diluted in PBS containing 10% FBS or serum samples diluted in PBS containing 0.02% NaN_3_ were added to the wells. The plates were incubated for 1 hour at RT on a shaker. Subsequently, the plates were washed 3 times with 200 µl PBS-Tween 20, and 100 µl of 2 mM adenosine in 20 mM Tris-HCl pH 6.8, 10 μM ZnCl_2_, 0.02% NaN_3_ was added. The plate was incubated at 37°C for 16-24 hours. The reaction was stopped by transferring 20 µl of the reaction mixture into a UV-transparent plate (Corning) containing 180 µl of water per well. The ratio of absorbance at 265 and 245 nm was detected on a Thermo Fischer Multiscan Go Reader. The ADA2 concentration in the samples was determined from a standard curve generated with the recombinant protein.

### Enzyme-linked immunosorbent assay (ELISA) for measurement of the active ADA1 concentration

ELISA plates (Greiner Bio-One) were coated overnight at 4°C with 100 µl of 2 µg/ml Streptavidin (New England BioLabs) in PBS with 0.02% NaN_3_. After washing the plates 3 times with 200 µl PBS-Tween 20 buffer and blocking with 2% BSA in PBS with 0.02% NaN_3_ for 1 hour, 100 µl of 1 µg/ml biotinylated rabbit anti-ADA1 polyclonal antibodies (Abcam) in 10% FBS with 0.02% NaN_3_ were added to the wells. The plate was incubated for 30 min at room temperature on a shaker. After washing the plates 3 times with 200 µl PBS-Tween 20, 100 µl of recombinant ADA1 standards diluted in PBS containing 0.5% BSA or saliva samples diluted in PBS with 0.02% NaN_3_ were added to the wells. The plates were incubated for 1 hour at RT on a shaker. Subsequently, the plates were washed 3 times with 200 µl PBS-Tween 20, and 100 µl of 0.3 mM adenosine in 20 mM Tris-HCl pH 7.3, 10 μM ZnCl_2_, 0.02% NaN_3_ was added. The plate was incubated at 37°C for 18-24 hours. The reaction was stopped by transferring 80 µl of the reaction mixture into a UV-transparent plate (Corning) containing 120 µl of water per well. The ratio of absorbance at 265 and 245 nm was detected on a Thermo Fischer Multiscan Go Reader. The ADA1 concentration in the samples was determined from a standard curve generated with the recombinant protein.

### Enzyme-linked immunosorbent assay (ELISA) for measurement of the total protein concentration of ADA2 in serum or saliva with HRP or ADA1 as detection enzymes

ELISA plates (Greiner Bio-One) were coated overnight at 4°C with 100 µl of 5 µg/ml rabbit anti-ADA2 polyclonal antibodies in PBS with 0.02% NaN_3_. After washing the plates 3 times with 200 µl PBS-Tween 20 buffer and blocking with 2% BSA in PBS with 0.02% NaN_3_ for 1 hour, 100 µl of recombinant ADA2 standards diluted in PBS containing 10% FBS or serum or plasma samples diluted in PBS containing 0.02% NaN_3_ were added to the wells. The plates were incubated for 1 hour at RT on a shaker. Subsequently, the plates were washed 3 times with 200 µl PBS-Tween 20 and 100 µl of 0.5 µg/ml affinity-purified biotinylated anti-ADA2 polyclonal antibodies diluted in PBS with 10% FBS were added to the wells. The plates were incubated for 1 hour at RT on a shaker and washed 3 times with 200 µl PBS-Tween 20. Then 100 µl of 1:2000 dilutions of HRP conjugated with Avidin (Abbkine) was added to the wells, and the plate was incubated for 30 min at room temperature on a shaker. Finally, the plate was washed 4 times with 200 µl PBS-Tween 20, and 100 µl of TMB substrate (eBioscience) was added. The reaction was stopped with 100 µl of 2M hydrochloric acid, and the plate was read on a Thermo Fischer Multiscan Go Reader at 450 and 570 nm. The total protein concentration of ADA2 in the samples was determined from a standard curve generated with wild-type recombinant protein. Alternatively, mouse ADA1 was used as a detection enzyme instead of HRP (26).

### Statistics

Statistical analysis was performed using Graphpad Prism and Excel software.

## Results

### ADA UV ELISA design

**Figure 1:**
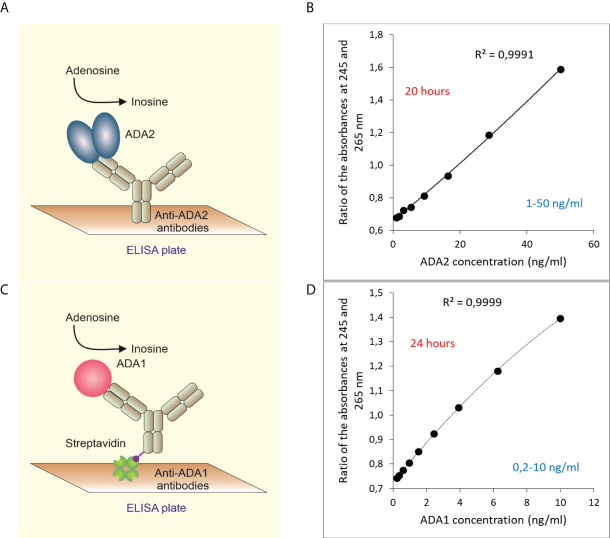
Quantitative detection of ADA2 or ADA1 in biological fluids based on ADA activity. **(A)** ADA2 is captured with rabbit polyclonal anti-ADA2 antibodies following the detection of ADA activity **(B)**. A typical standard curve was obtained after 20 hours of incubation with 2 mM adenosine. **(C)** ADA1 is captured with rabbit polyclonal anti-ADA1 antibodies on streptavidin-coated plates. Then adenosine is added to detect ADA activity. **(D)** A typical standard curve was obtained after 24 hours of incubation with 0.3 mM adenosine.

In a new, two-step ELISA assay, ADA2 is first selectively captured by anti-ADA2 rabbit polyclonal antibodies (**Figure 1A**, **Supplementary Figure 1A**). Then adenosine is added to the plate to detect the ADA activity of the enzyme bound to the antibodies. After the incubation time, the reaction is stopped by transferring the samples to a 96 wells UV-transparent plate. The ADA activity is proportional to the ratio of absorbances at 245 and 265 nm on a separate UV-transparent. This ratio increases following the conversion of adenosine to inosine (**Figure 1B**). The concentration of active ADA2 molecules is quantified using ADA2 standards. This assay is suitable for detecting ADA2 in serum or saliva when the concentration of ADA2 is within the assay’s detection limit (1 to 50 ng/ml of ADA2) or higher.

Similarly, ADA1 concentration in saliva can be detected using anti-ADA1 antibodies and human ADA1 standards (**Figure 1C**). In the experiments with saliva, we used streptavidin-coated ELISA plates to capture biotinylated anti-ADA1 antibodies (Materials and Methods). This setup allows for reducing the concentration of anti-ADA1 antibodies and using antibodies containing substances that interfere with the direct coating of the ELISA plate. As expected, the detection limit for ADA1 is lower than for ADA2 due to the higher activity of ADA1 at low adenosine concentration (**Figure 1D**). The catalytic parameters and activities of recombinant ADAs were measured using column chromatography (8, 26). Therefore, the concentration of ADA1 and ADA2 can be easily converted to enzymes activities at saturating concentrations of adenosine and optimal pH (**Table 1**). In addition, serum samples with known ADA2 concentrations could be used as the standards to simplify the method further.

### Sandwich ELISA design

**Figure 2:**
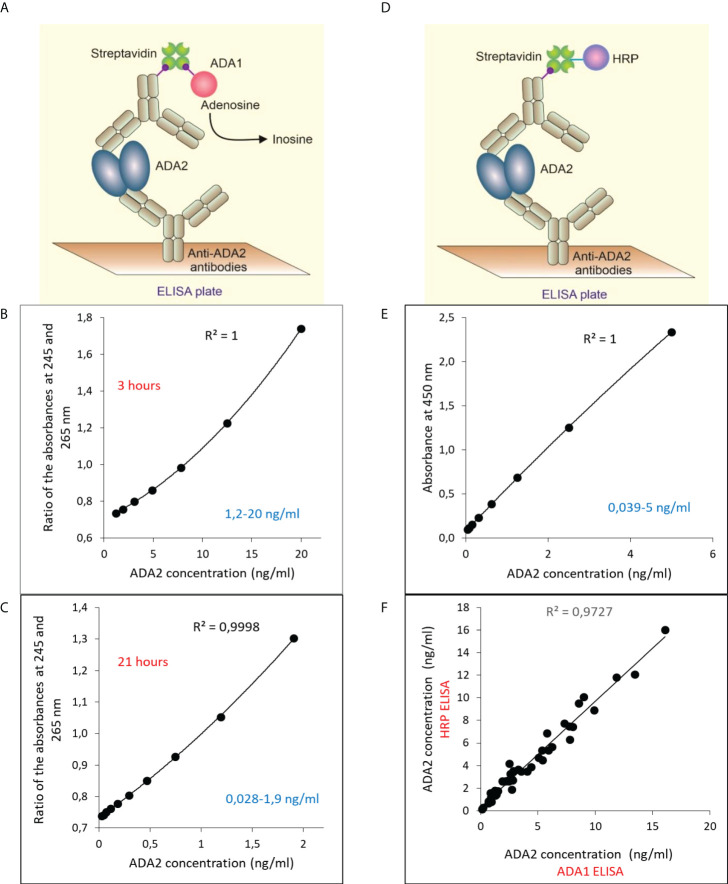
Sandwich ELISA for the detection of ADA2 with rabbit anti-ADA2 polyclonal antibodies. **(A)** Sandwich ELISA with mouse ADA1 (mADA1) as a detection enzyme. Typical standard curves were obtained with the ADA1 detection system after 3 hours **(B)** and 21 hours **(C)** of incubation with adenosine. **(D)** Sandwich ELISA with mouse HRP as a detection enzyme. A typical standard curve is shown in **(E)**. **(F)** Correlation between the results obtained for 47 saliva samples from children using mADA1 or HRP as detection enzymes.

To improve the sensitivity of ADA2 detection, we have developed a sandwich ELISA (**Figure 2**). Since ADA2 is a homodimer, we could use polyclonal antibodies to capture and detect the enzyme. We used ADA1 and horseradish peroxidase (HRP) as detection enzymes (**Figures 2A, B**). The use of ADA1 allows for detecting a broader range of ADA2 concentrations in the samples compared to HRP, and the ADA reaction is not sensitive to the presence of NaN_3_ (**Figures 2B, C**). However, HRP permits getting the results faster (**Figure 2E**), albeit at lower accuracy (26). As is shown in **Figure 2F**, the results obtained with both detection enzymes are very similar.

### Screening for ADA2 deficiency using serum samples

**Figure 3:**
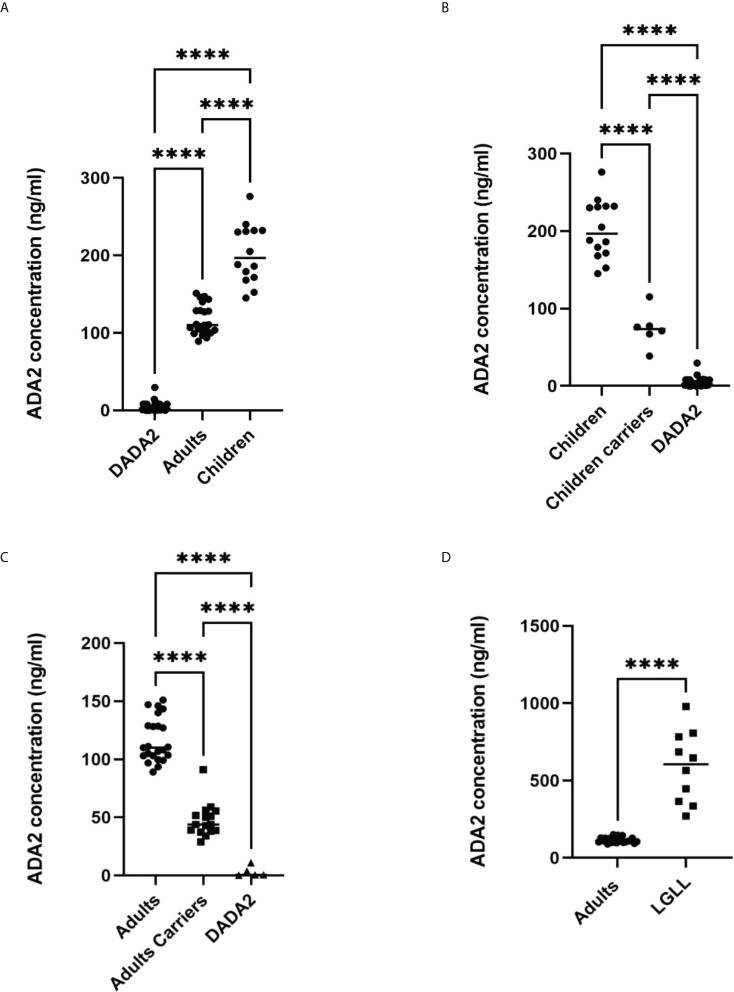
Analysis of ADA2 in serum of healthy donors and DADA2 patients using the method described in **Figure 1A**. **(A)** ADA2 concentration in serum samples from 14 healthy children, 23 healthy adults, and 32 DADA2 patients. **(B)** ADA2 concentration in serum samples from 14 healthy children, 6 children carriers, and 27 children DADA2 patients. **(C)** ADA2 concentration in serum samples from 23 healthy adults, 16 adults carriers, and 5 adult DADA2 patients. **(D)** ADA2 concentration in serum samples from 23 healthy adults and 10 LGLL patients. ****p<0.0001 according to one-way ANOVA test **(A–C)** or unpaired t-test **(D)**.

Previously, we used the method described in **Figure 1A** to diagnose ADA2 deficiency (9). **Figure 3** summarizes the published and new data obtained with serum samples from healthy adult and children controls and carriers of a pathogenic gene mutation in a single copy of ADA2 gene and DADA2 patients (27–29). As shown in **Figure 3A**, the concentration of ADA2 in the serum of healthy children is significantly higher than in healthy adults. However, the concentration of ADA2 in serum of DADA2 patients was much lower than ADA2 concentrations in both groups (**Figure 3A**) and, in some cases, below the assay’s detection limit. Thus this method allows diagnosing DADA2 with 100% sensitivity and specificity. Furthermore, children and adults with a pathogenic mutation in one copy of the ADA2 gene can be identified with the same specificity and sensitivity (**Figures 3B** **, C**). Furthermore, it was shown that some DADA2 patients have large granular lymphocytes (LGL) in the blood smear and bone marrow associated with the expansion of CD8+ T cells (27, 30). Therefore, we decided to analyze the plasma samples from patients with large granular lymphocytes leukemia (LGLL) to determine whether some cases of this rare disease are linked to DADA2. Surprisingly, it was found that all the serum samples from LGLL patients had a significantly higher concentration of ADA2 than the healthy controls. Therefore, ADA2 might be a new predictive marker for LGLL and an indicator of the positive outcome of the treatment (**Figure 3D**). The results of the ELISA assay had a 90% correlation with the results obtained using the ADA activity assay (8) (**Supplementary Figure 2**).

### Saliva collecting and storage

**Figure 4:**
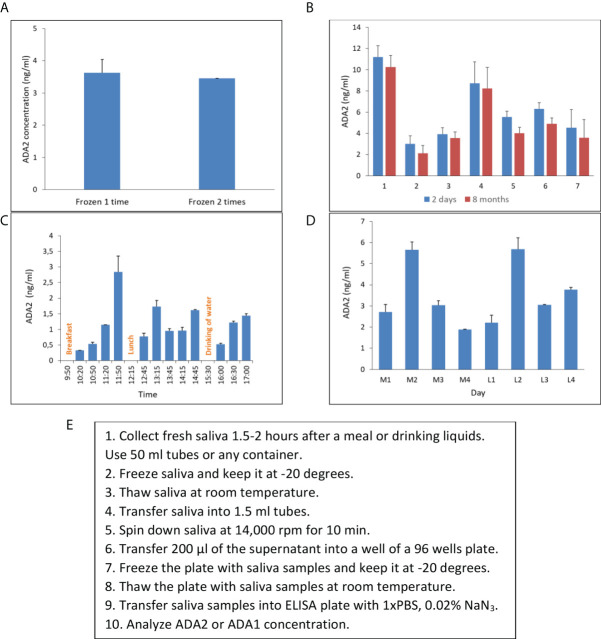
Saliva collecting and storage. **(A)** ADA2 concentration in a saliva sample frozen either one or two times. **(B)** The concentration of ADA2 in seven saliva samples that were stored for two days or eight months at -20°C. **(C)** Changes in ADA2 concentration in saliva during the day. Saliva samples were collected from a single donor at different times after breakfast, lunch, or drinking of liquids. **(D)** ADA2 concentration in saliva during four consequent days. The samples were taken in the morning on an empty stomach (M1-M4) and one hour after lunch (L1-L4). The data is the mean value of two replicates. **(E)** Saliva collecting and storage protocol. The ADA2 concentrations were detected according to the method described in **Figure 2**.

In the beginning, we had to design a standard protocol for saliva collection and processing. We aimed to keep the protocol for saliva sampling as simple as possible. Unstimulated saliva samples were collected in 50 ml tubes, transported to the lab on ice, and frozen at -20 degrees. After freezing and thawing, saliva becomes less dense and easy to pipette. Next, the samples were spanned in microcentrifuge tubes to remove bacteria and clarify saliva. The clarified supernatants were transferred into the 96-well plate and stored at -20 degrees to further analyze ADA2 or ADA1 concentration. Freezing and thawing the samples two times did not affect the concentration of ADA2 in saliva (**Figure 4A**). The concentration of ADA2 in the frozen samples was stable up to 8 months of storage (**Figure 4B**). To find the optimal time for saliva sampling, we checked the concentration of ADA2 in saliva at different times after meals or drinking water. As shown in **Figure 4C**, the concentration of ADA2 increased during the first hour after food or liquids intake. In the following period, the level of ADA2 in saliva was stabilized. Analysis of ADA2 in saliva samples obtained during the four consequent days revealed that the enzyme concentration in saliva samples collected in the morning on an empty stomach was similar to ADA2 concentration in saliva one hour after lunch (**Figure 4D**). Therefore, all the saliva samples in this work were collected within 1.5-2 hours after the food or drink (**Figure 4E**).

### ADA2 concentration in saliva for DADA2 diagnostics

**Figure 5:**
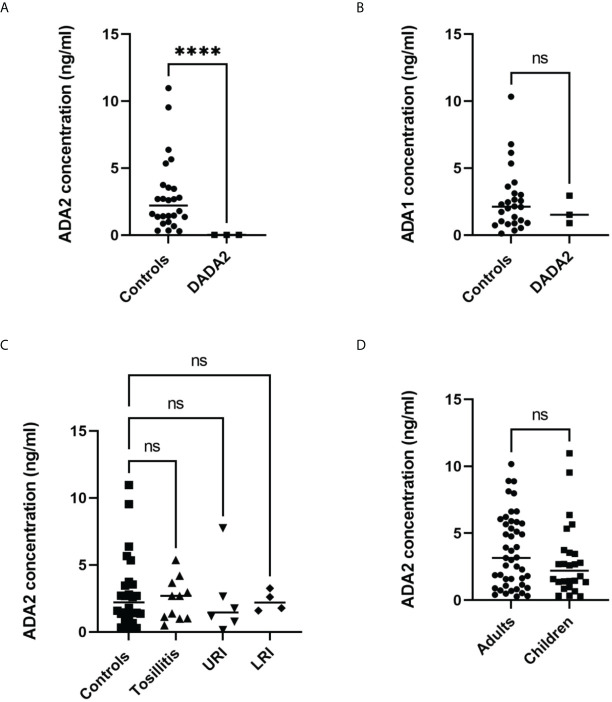
Analysis of saliva samples. **(A)** ADA2 concentration in saliva samples from DADA2 deficient children and healthy controls. ****p<0.0001 according to Welch’s t-test **(B)** ADA1 concentration in saliva samples from DADA2 deficient children and healthy controls. ****p<0.0001 according to unpaired t-test. **(C)** ADA2 concentration in saliva of healthy children, children with tonsillitis, upper respiratory infections (URI), and lower respiratory infections (LTI) **(D)** ADA2 concentration in saliva samples from adults and children. ****p<0.0001 according to unpaired t-test. The ADA2 and ADA1 concentrations were detected according to the method described in **Figure 1C** and **Figure 2**, respectively. ns, non-significant.

To measure ADA2 concentration in saliva, we used a sandwich ELISA described in **Figure 2**. The concentration of ADA2 in saliva varies significantly, and it is much lower than the concentration of ADA2 in serum (**Table 2**). Nevertheless, the concentration of ADA2 in DADA2 patients was dramatically reduced, and it was below the limit of detection (23 pg/ml). Thus, both saliva and serum could be used to diagnose DADA2 (**Table 3**). In contrast to ADA2, the concentration of ADA1 in the saliva of DADA2 patients was not significantly different from the control samples (**Figure 5B**). Likewise, in contrast to serum samples, the concentration of ADA2 was not significantly different between the control groups of children and adults (**Figure 5D**). In addition, children with tonsillitis and respiratory infections had a similar concentration of ADA2 to healthy controls (**Figure 5C**), suggesting that these infections do not affect the concentration of ADA2 in saliva.

**Table 2:** ADA2 concentration in saliva of different groups of patients and healthy controls.

Group	Number	Female	Male	Age	ADA2 (ng/ml)
**Children**					
Controls	26	12	14	8.3 (3–13)	2.9 (0.33-11.0)
DADA2	3	1	2	10 (8-12)	<0.023
Tonsillitis	11	3	8	6.3 (4-10)	2.42 (0.49-5.4)
Upper respiratory infections	6	5	2	6 (4-13)	2.37(0.14-7.74)
Lower respiratory infections	4		4	3-10	2.31(1.59-3.25)
**Adults controls**					
Controls	44	28	16	27.8 (20-45)	3.8 (0.28-10.2)
Confirmed cancer total	41	5	36	62.2 (47-80)	15.3 (0.3-61.1)
Confirmed cancer during therapy	24		24	61.0 (47-80)	16.5(0.3-61.1)
Confirmed cancer before treatment	17	5	12	63.8 (45-72)	13.9 (0.9-43.1)
Glottic carcinoma	16		16	60.9 (47-80)	11.1 (0.24-43.8)
Supraglottic carcinoma	7	7		58 (50-63)	5.1 (0.24-10.5)
Laryngeal cancer (unspecified)	10	3	7	66.1 (55-79)	20.3 (2.4-61.1)
Hypopharyngeal cancer	8		8	56.4 (57-64)	12.0 (3.1-26.5)
Tonsil carcinoma	3	1	2	61.3 (50-68)	29.0 (10.0-42.6)
				Mean (min-max)	Mean (min-max)

**Table 3:** Saliva and serum samples from DADA2 patients.

	Sex	Age	Mutation	ADA2(ng/ml)	ADA2(ng/ml)
DADA2	F	8	ADA2, c.C505G:p.R169G and c.G142A:p.G48R	2.02 ± 0.03	<0.023
DADA2	M	10	ADA2, c.C505G:p.R169G and c.G142A:p.G48R	1.79 ± 0.03	<0.023
Children controls				202 (145-276)	2.9 (0.33-11.0)
				serum mean (min-max)	saliva mean (min-max)

### ADA2 concentration in saliva from patients with head and neck cancer

**Figure 6:**
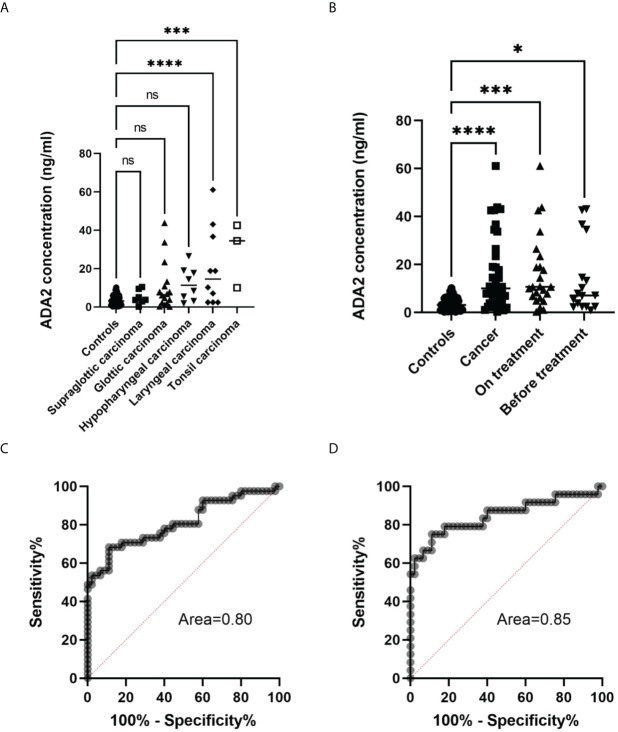
Analysis of saliva samples from head and neck cancer patients. **(A)** ADA2 concentration in saliva samples from healthy controls and patients with head and neck cancer types. ***p=0.0001, ****p<0.0001, according to one-way ANOVA test. **(B)** ADA2 concentration in saliva samples from healthy donors, all cancer patients, treated and not treated cancer patients. *p=0.01, ***p=0.0002, ****p<0.0001 according to one-way ANOVA test. **(C)** ROC curve constructed for all cancer samples **(D)** ROC curve constructed for samples from cancer patients undergoing treatment. The ADA2 concentrations were detected according to the method described in **Figure 2**. ns, non-significant.

The previous publication suggested that ADA activity in the saliva of preoperative patients with oral and laryngeal cancer is significantly lower compared to healthy controls (31). Therefore, we decided to analyze ADA2 concentration in saliva samples from head and neck cancer patients. As shown in **Table 2**, the mean concentration of ADA2 varied among patients with different types of head and cancer. While patients with supraglottic carcinoma had similar values of ADA2 to the control group, some of the patients from the other groups had a significantly higher concentration of ADA2 (**Figure 6A**). The highest difference in ADA2 concentration was detected in patients with laryngeal carcinoma and tonsil cancer groups. This result suggests that the concentration of ADA2 in saliva may depend on the tumor’s proximity to the oral cavity. Therefore, we narrowed further analysis with saliva samples from glottis, hypopharyngeal, laryngeal, and tonsil carcinoma (**Figure 6B**). The increase in ADA2 concentration was significantly higher in all patients with cancer, patients undergoing treatment, and untreated patients (**Figure 6B**). To demonstrate that ADA2 can be used as a predictive marker for active cancer, we constructed a ROC curve for ADA2 concentration in the samples from all cancer patients and the patients on treatment (**Figures 6C** **, D**). The area below the ROC curve was above 0.8 and 0.85, respectively, indicating that ADA2 is a good predictive marker for the studied types of cancer.

## Discussion

Clinical studies show that the activity of adenosine deaminases is a valuable biomarker for immune diseases and cancer. Several methods have been developed to measure ADA activity in biological samples, and published clinical studies utilize different assays (23, 24, 32). However, the presence of two adenosine deaminases with distinct catalytic parameters and the absence of a specific inhibitor of ADA2 makes the comparison of the results challenging. For example, the mean activity of ADA2 determined for the healthy controls in serum can vary from 3.85 IU/l to 17.0 IU/l depending on the study (14, 33). The difference in adenosine concentrations, pH of the buffers, and stability of the components of the activity assays could explain the discrepancy in the results.

However, screening a large population to diagnose diseases, rare genetic diseases requires the development of reproducible and simple methods. Here, we described several ELISA-based assays that could be used to screen patients and accurately measure the concentration of ADA2 and ADA1 in biological fluids. Using concentration instead of ADA activities allows unambiguously comparing the data from many clinical studies and improves the sensitivity of currently available methods of ADA activity detection. Our results show that quantitative assays could be successfully used to diagnose DADA2, LGLL, and follow-up the treatment of head and neck cancer.

The main advantage of our detection system is a combination of specific anti-ADA antibodies, which allow for selective capture of ADA2 or ADA1 from a biological sample, and the enzymatic ADA activity used to detect the enzyme bound to the antibodies (**Figure 1**; **Supplementary Figure 1**). Another advantage is the simplicity of detection of ADA activity based on the increase in the ratio of absorbances at 245 and 265 nm. The reaction of adenosine to inosine conversion is followed using reusable 96-well UV-transparent plates. The ADA reaction can be stopped and analyzed at any time point by transferring a sample from the ELISA to the UV plate. Hence it becomes possible to analyze samples with significant differences in ADA concentrations in a single analysis. As we have shown previously, the detection of ADA activity could be modified by adding enzymes that convert inosine to hydrogen peroxide and uric acid (26). In the presence of HRP, hydrogen peroxide reacts with a chemical substrate to produce colored or fluorescent substances. It allows for a reduction in the incubation times with adenosine and further increases the assay’s sensitivity. These ADA activity-based ELISA assays are ideal for measuring the concentration of ADA2 in serum. The results do not depend on the quality of the sample, such as contamination with red blood cells, mucosa, and insoluble particles. As shown in **Figure 3**, based on ADA2 concentration in serum samples, we differentiated the samples with confirmed LGLL, DADA2, and carriers of a pathogenic mutation in a single copy of the ADA2 gene. Since ADA2 is significantly increased in the LGLL, our data suggest that ADA2 deficient patients have another type of LGL leukemia. At the same time, ADA2 could be used as an additional marker to confirm LGLL and follow up on the treatment of this cancer (34).

To further increase the sensitivity of ADA2 detection, we developed an ELISA in which we used polyclonal antibodies to capture and detect ADA2 (**Figure 2**). The use of ADA1 instead of HRP as a detection enzyme permits analyzing the samples with a broad ADA2 concentration. This method could be used to analyze saliva samples where the concentration of ADA2 is much lower than serum (**Tables 2**, **3**). The utility of ADA2 as a biomarker in saliva has not been previously investigated. Therefore, we developed a saliva sampling and storage protocol (**Figure 4E**). The concentration of ADA2 in saliva changes during the day and after food intake in particular. Thus, the best time for sampling is at least after 1 hour after a meal of drinking (**Figure 4C**). The concentration of ADA2 in saliva samples remains stable for up to 8 months of storage at -20 degrees (**Figure 4B**). We used saliva samples from healthy donors to establish the concentration of ADAs in the control samples (**Table 2**, **Figure 5**). In contrast to serum samples (**Figure 3A**), there was no significant difference in ADA2 concentration in the control groups of children and adults. However, the concentration of ADA2 in the saliva samples of DADA2 patients was drastically reduced and found to be below the assay’s detection limit (**Figure 5A**). At the same time, the concentration of ADA1 in saliva from DADA2 patients remained normal (**Figure 5B**). Therefore, both saliva and serum samples can be used to diagnose DADA2 (**Table 2**). We also checked whether inflammation or respiratory diseases affect the concentration of ADA2 in saliva. However, analysis of saliva of children with tonsillitis and respiratory infections did not reveal any difference in ADA2 concentration (**Figure 5C**). Contrary, it was shown that the level of ADA2 in the saliva is increased in patients with Covid-19 (24). Therefore, it is still possible that the concentration of ADA2 may depend on the type of infection (viral or bacterial), and thus further investigations are required. It has to be also noted that saliva contains bacterial ADA, which may not be inhibited efficiently by EHNA, the inhibitor of human ADA1.

Some publications indicated that ADA activity is different in healthy controls and patients with oral cancers, though the results are contradictory (31, 35). Therefore, we collected and analyzed saliva from head and neck cancer patients (**Table 2**). Surprisingly, we found many cancer patients with increased ADA2 concentration in saliva. Careful analysis showed that the enzyme’s concentration depends on the type of cancer (**Figure 6A**). This data suggests that the location of the tumor relative to the oral cavity may affect the concentration of ADA2 in saliva. Hence, ADA2 could be a more suitable marker for glottic, hypopharyngeal, and tonsil carcinomas than supraglottic carcinoma. Indeed, the combined samples from patients with hypopharyngeal, glottic, and tonsil carcinomas have significantly higher ADA2 concentrations than healthy controls (**Figure 6B**). In addition, we noted the difference in ADA2 concentrations in saliva before the treatment and in the course of treatment. This observation could be explained by the difference in the severity of the disease (**Figure 6B**). Therefore, more studies are required to analyze ADA2 concentration at different stages of cancer to prove that ADA2 could be used as a predictive marker to diagnose specific head and neck cancers. Nevertheless, our results suggest that high ADA2 concentration in saliva could indicate persistent activation of the immune system in response to cancer (**Figures 6C, D**). Thus, ADA2, as a marker, could be helpful in follow-up the treatment of cancer patients where ADA2 concentration is significantly elevated.

Our data show that quantitative analysis of ADAs is a sensitive and straightforward way to screen a large population to reveal individuals with ADA deficiencies, immune disorders, and cancer.

## Data availability statement

The original contributions presented in the study are included in the article/**Supplementary Material**. Further inquiries can be directed to the corresponding author.

## Ethics statement

The studies involving human participants were reviewed and approved by Guangzhou Women and Children’s Hospital. Written informed consent to participate in this study was provided by the participants’ legal guardian/next of kin.

## Author contributions

AZ designed, supervised the research, analyzed the data, and wrote the manuscript. AZ, WWL, and LD performed the experiments. WBL, LH, FC, WWL, TL, and QZ collected the patient’s data and samples. All authors contributed to the article and approved the submitted version.

## Funding

This work was supported by Guangzhou Women and Children’s Hospital, Guangzhou Science and Technology Project 202201011494 to LD, and a grant 256053 from the Finnish Academy to AZ.

## Acknowledgments

The authors wish to thank the staff of the Institute of Pediatrics for participating in the study.

## Conflict of interest

The authors declare that the research was conducted in the absence of any commercial or financial relationships that could be construed as a potential conflict of interest.

## Publisher’s note

All claims expressed in this article are solely those of the authors and do not necessarily represent those of their affiliated organizations, or those of the publisher, the editors and the reviewers. Any product that may be evaluated in this article, or claim that may be made by its manufacturer, is not guaranteed or endorsed by the publisher.
