# Supplementary material for: ELISA based assays to measure adenosine deaminases concentration in serum and saliva for the diagnosis of ADA2 deficiency and cancer
Source: Front Immunol. 2022 Jul 28;13:928438. doi: 10.3389/fimmu.2022.928438 (PMC9366848; doi:10.3389/fimmu.2022.928438)
Supplement: Supplementary file 1 [file DataSheet_1.pdf]

Supplementary material

Supplementary Figure 1. Specificity of the anti-ADA2 and anti-ADA1 antibodies. (A) A standard curve was obtained with rabbit polyclonal anti-ADA2 antibodies and either ADA1 or ADA2 standards (see Figure 2E). (B) A standard curve was obtained with rabbit polyclonal anti-ADA1 antibodies and either ADA1 or ADA2 standards (see Figure 1D).

Supplementary Figure 2. Correlation between the results obtained for 10 serum samples from patients with LGLL using ELISA assay (Figure 1A) and ADA2 activity in the same samples. The ADA2 activity in the serum of LGLL patients was determined using an assay described by Zavialov and Engstrom (1). ADA1 inhibitor EHNA was added into the incubation mixture to a final concentration of 0.1 mM.

A

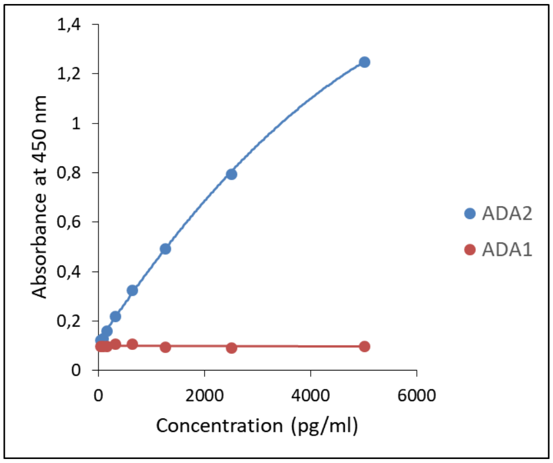

B

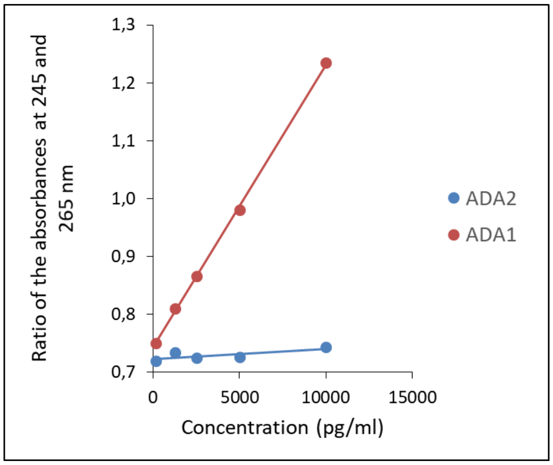

Supplementary Figure 1

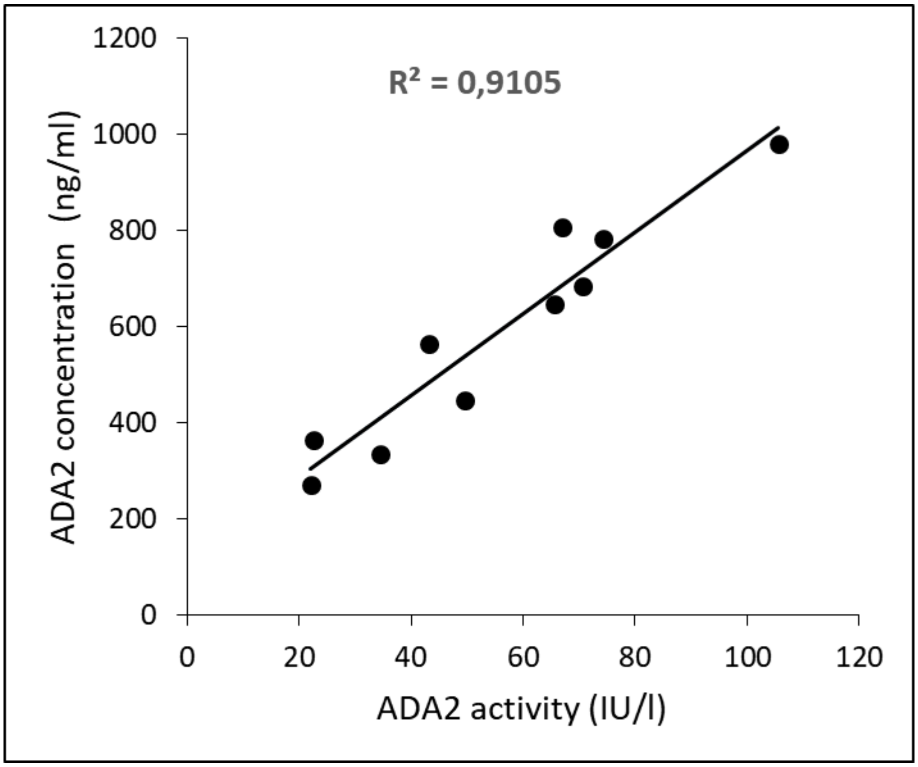

Supplementary Figure 2

References:

1.Zavialov Andrey V, Engström Å. Human ADA2 belongs to a new family of growth factors with adenosine deaminase activity. Biochemical Journal. 2005;391(Pt 1):51-7.
